# Discovery of Kuraridin as a Potential Natural Anti-Melanogenic Agent: Focusing on Specific Target Genes and Multidirectional Signaling Pathways

**DOI:** 10.3390/ijms252011227

**Published:** 2024-10-18

**Authors:** Subin Jeon, Kumju Youn, Mira Jun

**Affiliations:** 1 Department of Health Science, The Graduate School, Dong-A University, Nakdong-daero 550 beon-gil, Saha-gu, Busan 49315, Republic of Korea; 1939584@donga.ac.kr; 2 Department of Food Science and Nutrition, Dong-A University, Nakdong-daero 550 beon-gil, Saha-gu, Busan 49315, Republic of Korea; kjyoun@dau.ac.kr; 3 Center for Food & Bio Innovation, Dong-A University, Nakdong-daero 550 beon-gil, Saha-gu, Busan 49315, Republic of Korea

**Keywords:** anti-melanogenesis, depigmenting agent, kuraridin, network pharmacology, c-KIT, ERK1/2, PKC

## Abstract

Abnormal melanogenesis upon UV exposure causes excessive oxidative stress, leading to hyperpigmentation disorders. As a key rate-limiting enzyme in melanogenesis, tyrosinase is considered a primary target for depigmenting agents. *Sophora flavescens* is used as a food and in traditional medicine as a valuable source of prenylated flavonoids. The present study aimed to elucidate the anti-melanogenic effect and potential mechanism of kuraridin, one of the major prenylated flavonoids. Kuraridin showed anti-tyrosinase activity with an IC_50_ value in the nanomolar range, superior to that of kojic acid, a positive control. It significantly reduced tyrosinase activity with the least cytotoxicity, suppressing melanogenesis in α-MSH-induced B16F10 cells. Furthermore, kuraridin considerably reduced melanogenesis in a 3D human skin model. To elucidate the anti-melanogenic mechanism of kuraridin, target genes (KIT, MAP2K1, and PRKCA) and pathways (c-KIT and ETB-R pathways) were identified using network pharmacology. KIT and MAP2K1 are simultaneously involved in the c-KIT cascade and are considered the most important in melanogenesis. PRKCA acts directly on MITF and its downstream enzymes through another pathway. Docking simulation showed strong interactions between kuraridin and c-KIT, ERK1/2, and PKC encoded by target genes. Overall, the present study showed kuraridin to be a novel natural anti-melanogenic agent in hyperpigmentation disorders.

## 1. Introduction

Melanin, the primary pigment responsible for skin color, plays a critical role in scavenging free radicals generated in the cytoplasm and protecting the skin from ultraviolet (UV) radiation [1]. It is synthesized in melanocytes during melanogenesis and is mediated by several melanogenic enzymes and biochemical events. This process is completed by the transfer of melanin-loaded melanosomes to adjacent keratinocytes in the human epidermis [2]. However, prolonged exposure to UV radiation causes the accumulation of abnormal melanin, resulting in hyperpigmentation. Skin whitening is a widespread practice among some ethnic groups, particularly in Asia, Africa, and the Middle East, due to a complex interplay of cultural, social, political, and psychological factors [3], and lighter skin color is considered a critical beauty standard in these regions. Global market research shows that approximately 28% of the world’s population has undergone skin whitening at least once, and spending on the skin-whitening products market is expected to reach $16.1 billion by 2030, indicating continued expansion of the market potential [4,5]. Anti-melanogenic agents are also used to treat skin disorders caused by hyperpigmentation, such as melasma, freckles, lentigines, and post-inflammatory hyperpigmentation.

UV radiation stimulates melanogenesis through direct effects on melanocytes and indirect impact on keratinocytes, which release melanogenic components [6]. In response to UV-induced DNA damage in keratinocytes, alpha-melanocyte-stimulating hormone (α-MSH) is released, which binds to the melanocortin 1 receptor (MC1R), leading to the accumulation of melanin-containing melanosomes in melanocytes. Specifically, α-MSH binding promotes the expression and activity of key melanogenesis enzymes through the adenyl cyclase (AC)/cyclic AMP (cAMP)/protein kinase A (PKA) signaling pathway [7]. Melanin biosynthesis begins with the important action of tyrosinase, which catalyzes the *o*-hydroxylation of monophenols (L-tyrosine) to *o*-diphenols (L-DOPA) using molecular oxygen. Subsequently, this enzyme further oxidizes L-DOPA to dopachromes, which spontaneously polymerize to form melanin. This dual oxidation process by tyrosinase initiates the melanogenesis and the remainder of the reaction proceeds non-enzymatically under physiological conditions [8].

Since tyrosinase is a major enzyme involved in melanogenesis, many studies have been devoted to tyrosinase inhibition. Many tyrosinase inhibitors are derived from natural sources, such as kojic acid, vitamin C, cysteine, and arbutin. Despite considerable efforts to develop tyrosinase inhibitors, they have been limited because of skin permeability, stability, and safety concerns [9,10,11]. Hydroquinone, one of the most commonly used skin-lightening agents in clinical practice, is also a potent tyrosinase inhibitor. However, its use is recommended with caution because of poor skin penetration and irritant dermatitis [11]. Therefore, identifying new tyrosinase inhibitors with high efficacy and fewer side effects is important for the prevention of hyperpigmentation.

*Sophora flavescens* AITON (Leguminosae) is a perennial shrub found in the wild that is cultivated throughout Northeast Asia. The dried root of *S. flavescens* is a functional food and traditional herbal medicine used extensively for the treatment of antipyretic, hepatitis, analgesic, and gastrointestinal disorders in China, Japan, India, and Korea [12]. Previous studies have reported that alkaloids and flavonoids are the major bioactive groups in *S. flavescens*, and more than 60 alkaloid and 120 flavonoid compounds have been isolated to date [13]. In particular, *S. flavescens* contains large numbers of prenylated flavonoids, a unique class of flavonoids characterized by the substitution of the flavonoid ring system with a prenylated group, which increases lipophilicity. To date, approximately 70 prenylated flavonoids have been isolated and identified from *S. flavescens*; these exert various biological effects, including anti-inflammatory, anti-Alzheimer’s disease, anti-diabetic, and antitumor effects [14].

In an effort to identify active anti-melanogenic agents from nature, three major prenylated flavonoids from *S. flavescens*, kuraridin, kushenol A, and kurarinol, were chosen to determine their anti-melanogenic properties and regulatory mechanisms. First, the inhibitory properties of tyrosinase, the key rate-limiting enzyme in melanin synthesis, were evaluated using both L-tyrosine and L-DOPA. Second, the mode of interaction of the three compounds with the enzyme was thoroughly investigated using a kinetics study, and their safety and skin penetration were predicted using in silico pharmacokinetics. The inhibitory effects of the active compounds on melanin synthesis were validated in B16F10 melanoma cells and a three-dimensional (3D) human skin model. Finally, network pharmacology and molecular docking simulations were used to provide new insights into the anti-melanogenesis regulatory mechanism of the compound as part of the ongoing work to discover potent anti-melanogenesis agents.

## 2. Results

### 2.1. Anti-Tyrosinase Activities of Kuraridin, Kushenol A, and Kurarinol with L-Tyrosine and L-DOPA Substrates

**Figure 1:**
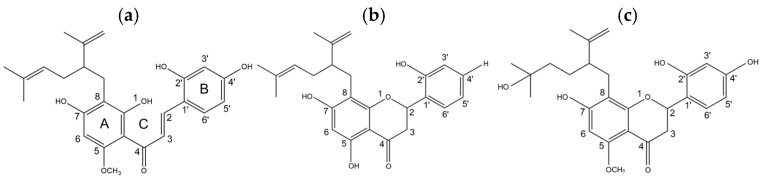
Chemical structures of *S. flavescens*: (**a**) kuraridin, (**b**) kushenol A, and (**c**) kurarinol.

**Figure 2:**
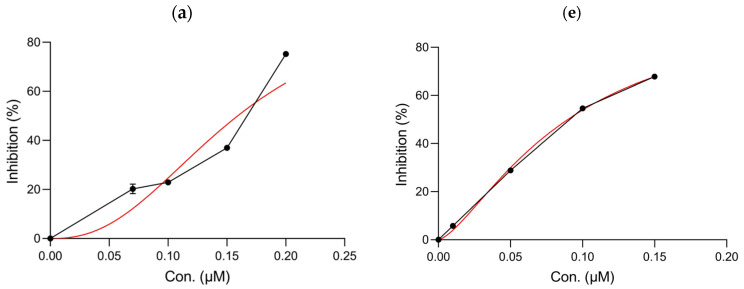

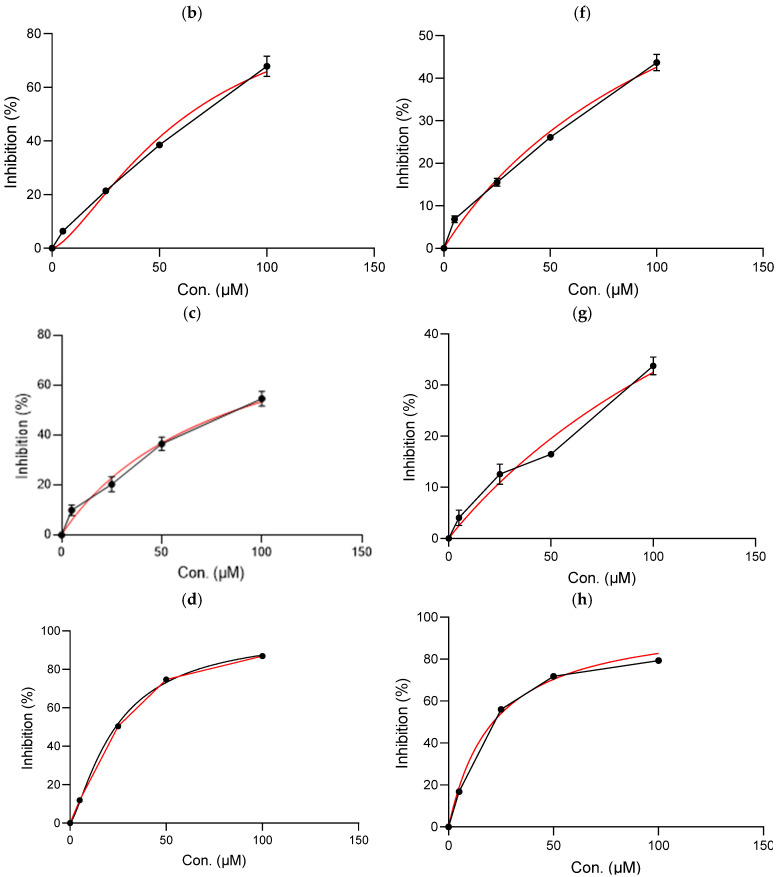
Inhibitory properties of kuraridin, kushenol A, and kurarinol on tyrosinase activity. The anti-tyrosinase activities using L-tyrosine were (**a**) kuraridin, (**b**) kushenol A, (**c**) kurarinol, and (**d**) kojic acid, and the anti-tyrosinase activities using L-DOPA were (**e**) kuraridin, (**f**) kushenol A, (**g**) kurarinol, and (**h**) kojic acid. Black and red lines represent IC_50_ values and the trend values, respectively.

The chemical structures of kuraridin, kushenol A, and kurarinol are shown in Figure 1. To clarify the inhibitory effects of the three compounds on tyrosinase activity, the monophenolase activity was first determined using L-tyrosine as the substrate. As shown in Figure 2 and Table 1, kuraridin showed the most potent anti-monophenolase activity, with an IC_50_ value of 0.16 μM, in which the inhibitory effect was 148 times stronger than that of kojic acid, one of the most potent tyrosinase inhibitors in nature. In contrast, kushenol A and kurarinol showed moderate inhibitory effects.

Second, dopachrome formation was measured using L-DOPA as a substrate to confirm the inhibitory effects of the three compounds on tyrosinase diphenolase activity. It is noteworthy that kuraridin (IC_50_, 0.04 μM) had superior diphenolase inhibitory activity to that of the other compounds, kushenol A and kurarinol (IC_50_ > 100 μM), and was at least 530-fold more potent than kojic acid. Furthermore, the remarkable inhibitory effect of kuraridin was much stronger than the levels of well-known tyrosinase inhibitors from natural and synthetic sources such as arbutin, quercetin, resveratrol, and thiazolidinedione analogs, with IC_50_ values in the range of 1–200 μM [16].

### 2.2. Enzyme Kinetic Analysis of Prenylated Flavonoids Against Tyrosinase

**Figure 3:**
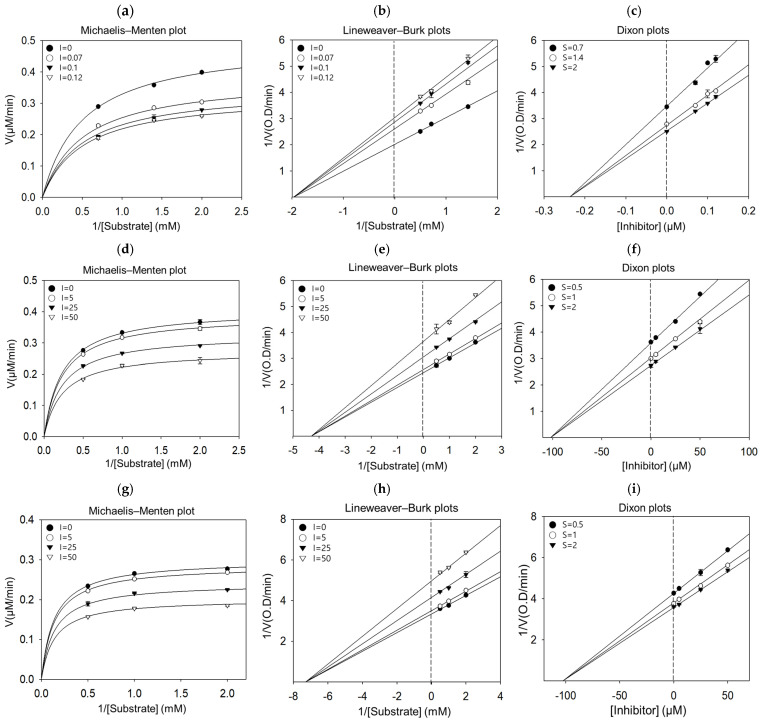

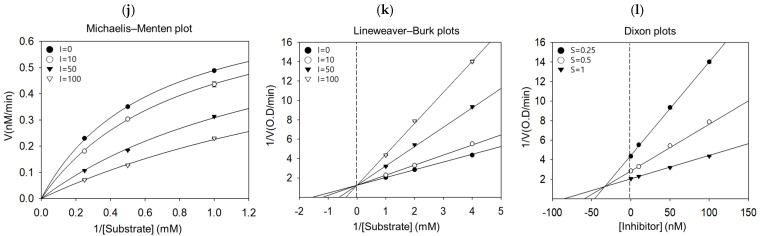
The kinetics of kuraridin, kushenol A, and kurarinol against tyrosinase activity using L−tyrosine and L−DOPA. Michaelis−–Menten plots for anti-tyrosinase activity by (**a**) kuraridin, (**d**) kushenol A, and (**g**) kurarinol in the presence of L−tyrosine. Lineweaver–Burk plots for anti-tyrosinase activity by (**b**) kuraridin, (**e**) kushenol A, and (**h**) kurarinol in the presence of _L_-tyrosine. Dixon plots for anti-tyrosinase activity by (**c**) kuraridin, (**f**) kushenol A, and (**i**) kurarinol in the presence of L−tyrosine. (**j**) Michaelis–Menten plot, (**k**) Lineweaver–Burk plot, and (**l**) Dixon plot for anti-tyrosinase activity by kuraridin in the presence of _L_-DOPA.

The kinetic characteristic of the oxidation of L-tyrosine catalyzed by tyrosinase in the presence of three compounds with potent inhibitory activity (IC_50_ < 100 μM) was investigated. As shown in Figure 3a–i and Table 1, all compounds were non-competitive inhibitors of monophenolase activity, indicating that they bind to the allosteric site rather than the substrate-binding site in tyrosinase, leading to a reduction in enzyme activity while maintaining the substrate affinity of the enzyme. Furthermore, the Ki values of kuraridin, kushenol A, and kurarinol were 0.24, 102.3, and 100.6 μM, respectively, indicating that kuraridin had the highest binding affinity with tyrosinase, which is consistent with its inhibitory property.

As depicted in Figure 3j,k and Table 1, graphical analysis of the Michaelis–Menten and Lineweaver–Burk plots of kuraridin, the only tyrosinase inhibitor with L-DOPA, showed competitive inhibition, as treatment with the compound resulted in double reciprocal straight-line plots with different slopes that increased the y-axis at the same point. These results suggest that kuraridin competitively interacts with substrate-binding sites to block tyrosinase activity when co-treated with varying concentrations of L-DOPA. In addition, kuraridin exhibited competitive inhibition against diphenolase activity (Ki, 0.27 μM), as shown in the Dixon plot (Figure 3l).

### 2.3. Pharmacokinetic Profiles of Prenylated Flavonoids for Bioavailability

Information on pharmacokinetic and toxicological properties is critical for understanding both the biological efficacy exerted on a target tissue and the safety of anti-melanogenic agents. The development of potent candidates requires considerable attention to their pharmacokinetic properties, such as intestinal absorption, safety, and skin permeability, which define the rate at which a molecule penetrates the stratum corneum. As shown in Table 2, the skin permeability value was below a threshold of −2.5, indicating low stratum corneum permeability. In contrast, the three compounds had skin permeability values greater than −2.5, suggesting that they can penetrate the stratum corneum to reach melanocytes. Moreover, these compounds exhibit high levels of human intestinal absorption, with >70% absorption in the gastrointestinal tract. In the present study, in silico prediction of the toxicity of the three prenylated flavonoids showed no signs of skin sensitization, minnow toxicity, mutagenicity, hepatotoxicity, or carcinogenicity. These reliable in silico findings on adverse indices will contribute to the safety of future kuraridin-containing products, such as skin-whitening cosmetics and depigmentation agents for hyperpigmentation disorders.

### 2.4. Inhibitory Effect of Kuraridin on Melanogenesis in B16F10 Cell

**Figure 4:**
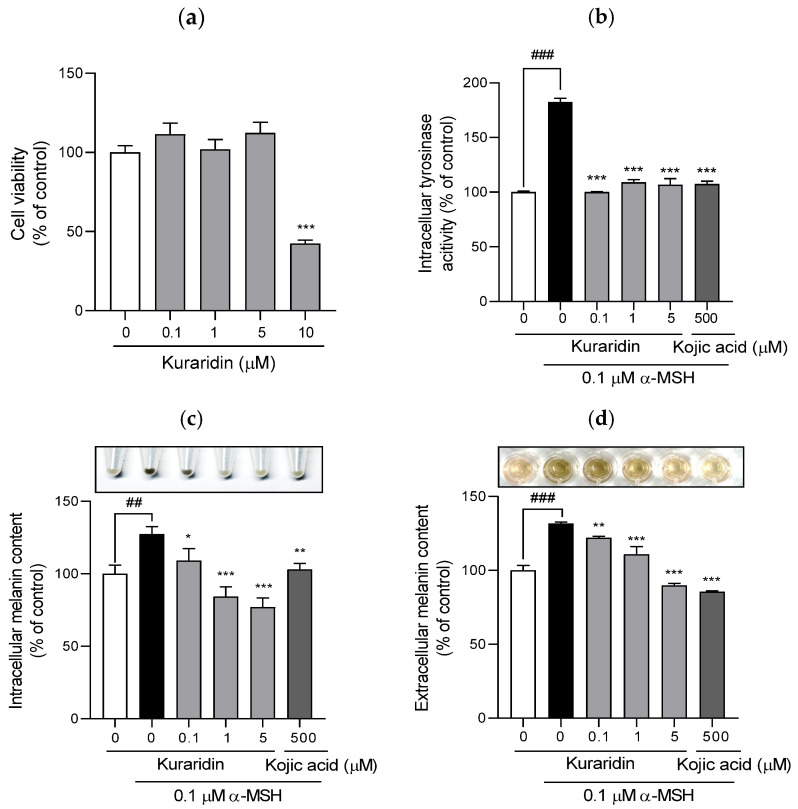
Effect of kuraridin on the viability, tyrosinase activity, and intracellular and extracellular melanin contents of B16F10 cells. (**a**) Cell viability was determined by the CCK assay. Cultured B16F10 cells were pretreated with kuraridin (0.1, 1, and 5 μM) for 72 h. (**b**) Cells were collected and centrifuged to obtain pellets. The pellets were dissolved in 1 N NaOH, and the relative amount of melanin was determined by measuring the absorbance at 405 nm. (**c**,**d**) After treatment under the same conditions used for the determination of melanin synthesis, cells were collected and lysed. Tyrosinase activity was measured using L-DOPA as a substrate. Cell viability, melanin contents, and tyrosinase activity in control cells were considered 100%. Values are expressed as the mean ± SD in at least three independent experiments. ^###^ *p* < 0.001 and ^##^ *p* < 0.01, as compared with the control group; *** *p* < 0.001, ** *p* < 0.01 and * *p* < 0.05 as compared with α-MSH only.

As kuraridin exhibited the highest tyrosinase inhibition at the cell-free level, its inhibitory effect on tyrosinase activity and melanin production was further investigated in B16F10 melanoma cells. As shown in Figure 4a, a concentration range of 0.1–5 μM kuraridin without cytotoxicity was selected for further experiments. When cells were treated with α-MSH, intracellular tyrosinase activity increased 1.8-fold compared with that in the control group (*p* < 0.001, Figure 4b). In contrast, kuraridin significantly suppressed tyrosinase activity at all tested concentrations (*p* < 0.001). Notably, the inhibition of intracellular tyrosinase activity by kuraridin reached control levels even at the lowest concentration (0.1 μM).

As shown in Figure 4c,d, stimulation with α-MSH noticeably increased the extracellular and intracellular melanin content to approximately 132% and 127%, respectively. However, kuraridin treatment significantly attenuated both the extracellular and intracellular melanin production in B16F10 melanoma cells in a dose-dependent manner. In addition, the compound at 5 μM inhibited α-MSH-induced increases in extracellular melanin similar to kojic acid at 500 μM. Treatment of α-MSH-treated cells with 1 µM of kuraridin resulted in a 15% lower intracellular melanin content than the untreated control.

### 2.5. Anti-Melanogenic Effect of Kuraridin in 3D Human Skin Model

Although the anti-melanogenic activity of kuraridin was demonstrated in vitro, it is difficult to ensure efficacy in humans as there are many confounding factors involved in verifying the inhibition of melanogenesis by kuraridin in human skin [17]. Therefore, the whitening property of kuraridin was further investigated in a 3D human skin reconstruction model. Composed of human primary keratinocytes and melanocytes, the model is morphologically identical to human skin and exhibits an in vivo histological morphology characterized by a multilayered, stratified, and pigmented epidermis.

**Figure 5:**
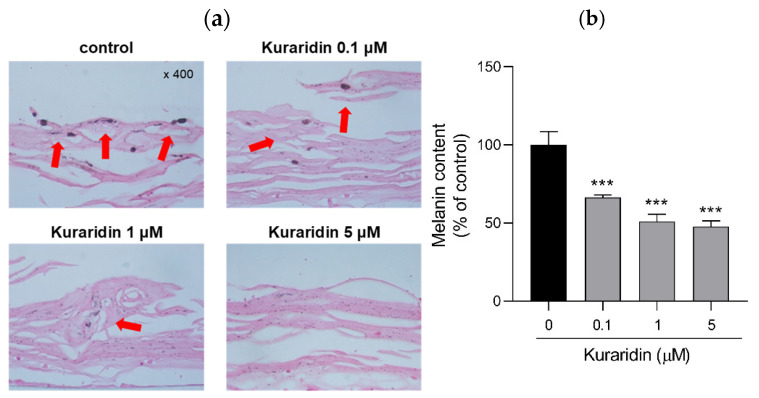
Anti-melanogenic effect of kuraridin in reconstituted 3D human skin model. Human skin tissues were pre-treated with kuraridin. (**a**) Representative images of Fontana–Masson-stained paraffin sections treated with control, 0.1 μM kuraridin, 1 μM kuraridin, and 5 μM kuraridin. (**b**) Melanin accumulation was quantified. *** *p* < 0.001 compared with the control group. Red arrows indicate the melanin deposition.

As shown in Figure 5a, melanin contents in the epidermis were significantly reduced after treatment with kuraridin at all concentrations (*p* < 0.001), as shown by Fontana–Masson-stained tissue sections. Interestingly, kuraridin at 0.1, 1, and 5 μM doses significantly reduced melanin content by 66.41 ± 1.54%, 54.07 ± 4.7%, and 47.87 ± 3.6%, respectively (Figure 5b).

### 2.6. Targets of Kuraridin on Anti-Melanogenesis Using Network Pharmacology

Because the traditional “one gene, one target, one disease” paradigm has been an obstacle in many aspects of drug discovery strategies for complex diseases, the “multi-target, multi-effect, complex disease” approach using network pharmacology, which designs novel leads that target multiple proteins, is considered the next paradigm in drug discovery [18]. Kuraridin exhibits a potent anti-melanogenic effect by suppressing tyrosinase activity; however, its molecular mechanism of action in melanogenesis remains unclear. In the next step in this study, network pharmacology was used to analyze multiple targets of the compound and to highlight the multidirectional regulation of signaling pathways that will help in understanding the prevention and treatment of hyperpigmentation.

**Figure 6:**
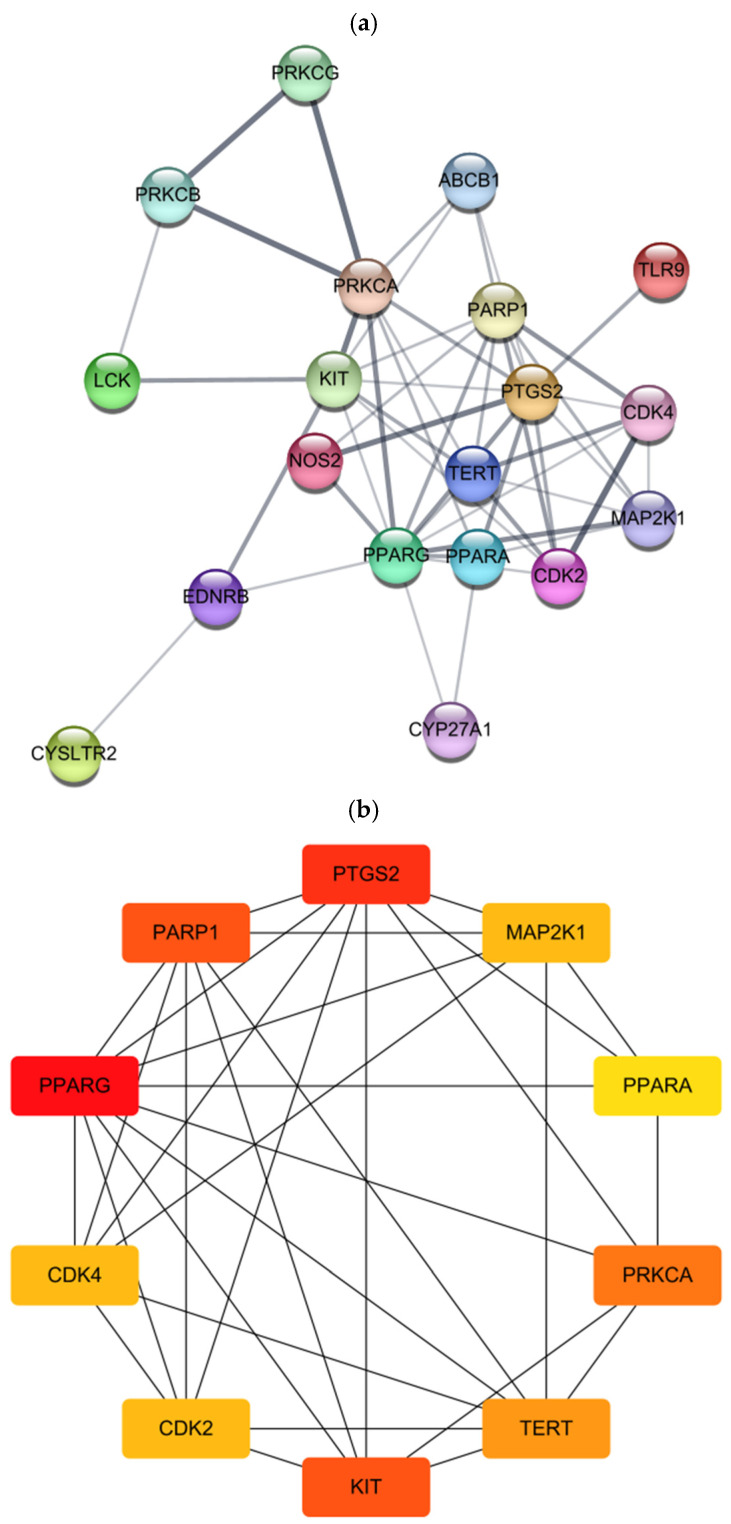

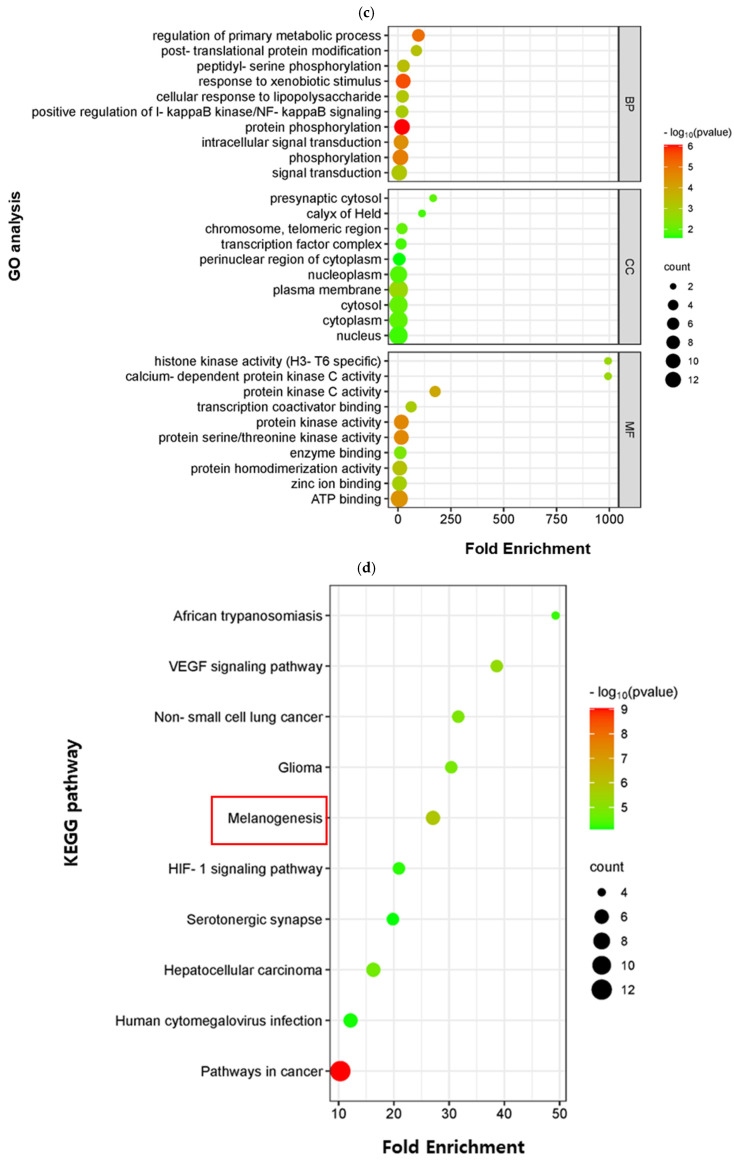

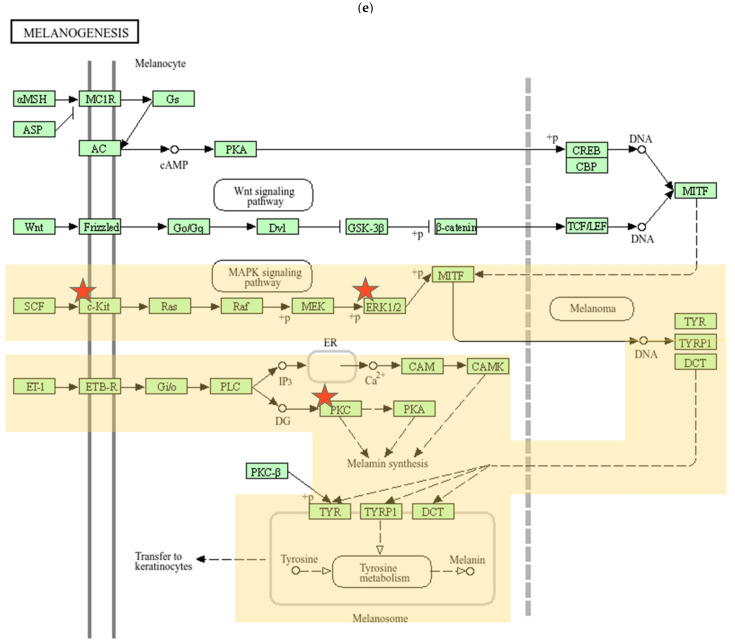
The protein–protein interaction (PPI) network of kuraridin in melanogenesis targets. (**a**) The PPI network diagram was constructed by STRING. Nodes (circles) and edges (lines) represent the target genes and the interaction of genes, respectively. (**b**) The top ten hub genes with a higher degree of connectivity from the PPI network. (**c**) Gene ontology (GO) and (**d**) Kyoto Encyclopedia of Genes and Genomes (KEGG) pathway analysis using the DAVID database. The GO terms were subdivided into biological process (BP), cell composition (CC), and molecular function (MF). In a KEGG pathway plot, the x-axis represents the gene ratio; the y-axis represents the enrichment pathway; the size of the dot represents the number of genes; the color of the dot represents the level of the *p*-value. Red frame indicates strongly associated pathway. (**e**) Predicted anti-melanogenesis-related genes (red star) and signaling pathways (yellow highlight) of kuraridin by KEGG analysis.

In the first step of the network pharmacology analysis, 513 targets for melanogenesis and 102 targets for kuraridin were identified using GeneCards and SwissTargetPrediction, respectively. Protein–protein interactions (PPI) for 19 overlapping genes between melanogenesis and kuraridin were constructed in the second step, resulting in 19 nodes (circles) and 50 edges (connecting lines; Figure 6a). Third, among the nodes of kuraridin, the top 10 targets with a high degree of connectivity were selected as hub genes as follows: PPARG, PTGS2, KIT, PARP1, PRKCA, TERT, CDK2, CDK4, MAP2K1, and PPARA (Table 3 and Figure 6b).

Gene Ontology (GO) enrichment analysis was used to identify the molecular functions (MF), cellular components (CC), and biological processes (BP) associated with the target genes of kuraridin in melanogenesis. A total of 14 CC, 24 MF, and 69 BP were enriched in the GO analysis (), and the top 10 were selected after ranking by *p*-value correction. As shown in Figure 6c, it was found that kuraridin-melanogenesis target genes were involved in MF such as protein kinase activity, protein serine/threonine kinase activity, and ATP binding; exerted CC, including membrane, cytosol, chromosome, and telomeric region; and participated in BP, including protein phosphorylation, response to xenobiotic stimulus, and regulation of metabolic processes.

Kyoto Encyclopedia of Genes and Genomes (KEGG) enrichment analysis was performed to elucidate the signaling pathways of proteins encoded by target genes. The results showed that kuraridin was most strongly associated with the melanogenesis pathway but not with the cancer pathway (Figure 6d). Moreover, three hub genes, KIT, protein kinase C alpha (PRKCA), and mitogen-activated protein kinase 1 (MAP2K1), were found to be closely related to the melanogenesis pathway, suggesting that these results further narrowed the scope of research on the anti-melanogenic mechanism of kuraridin. Figure 6e represents the predicted anti-melanogenesis-related target genes and kuraridin-signaling pathways. Kuraridin was shown to regulate two melanogenic signaling pathways such as tyrosine-protein kinase Kit receptor (c-KIT) and endothelin receptor type B (ETB-R), among four different melanogenic signaling pathways.

First, kuraridin directly acts on the KIT gene, which encodes a c-KIT that binds to the specific ligand stem cell factor (SCF), leading to receptor autophosphorylation and activation. The activated receptor phosphorylates RAS, which further activates the Raf-1 and MAPK signaling pathways. Interestingly, this compound was also directly linked to MAP2K1, which encodes extracellular signal-regulated kinase (ERK) ½, a vital member of the c-KIT signaling pathway. Activated ERK1/2 triggers the phosphorylation of microphthalmia transcription factor (MITF) at serine 73, which is then targeted for degradation. It is important to note that kuraridin directly affects two factors in the c-KIT pathway, the main pathway for melanogenesis. Kuraridin affects melanogenesis by acting on PRKCA in the ETB-R signaling pathway. The binding of ETB-R to endothelin 1 (ET-1) activates phospholipase Cc (PLCc) and increases phosphatidylinositol 4,5-bisphosphate (PIP2) hydrolysis, generating inositol trisphosphate (IP3) and diacylglycerol (DG). Kuraridin is highly associated with PRKCA, which encodes a PKC protein directly activated by the DG. The PKC-mediated pathway in melanogenesis is not well understood; however, it appears to involve the activation of PKA-mediated MITF upregulation, which in turn allows the subsequent transcription of melanogenic enzymes such as tyrosinase. To the best of our knowledge, this is the first study to demonstrate the potent anti-melanogenic effects of kuraridin mediated through multidirectional regulation of the melanogenic signaling pathway, suggesting its potential as an ideal skin-whitening agent.

### 2.7. Molecular Docking Validation of Kuraridin

**Figure 7:**
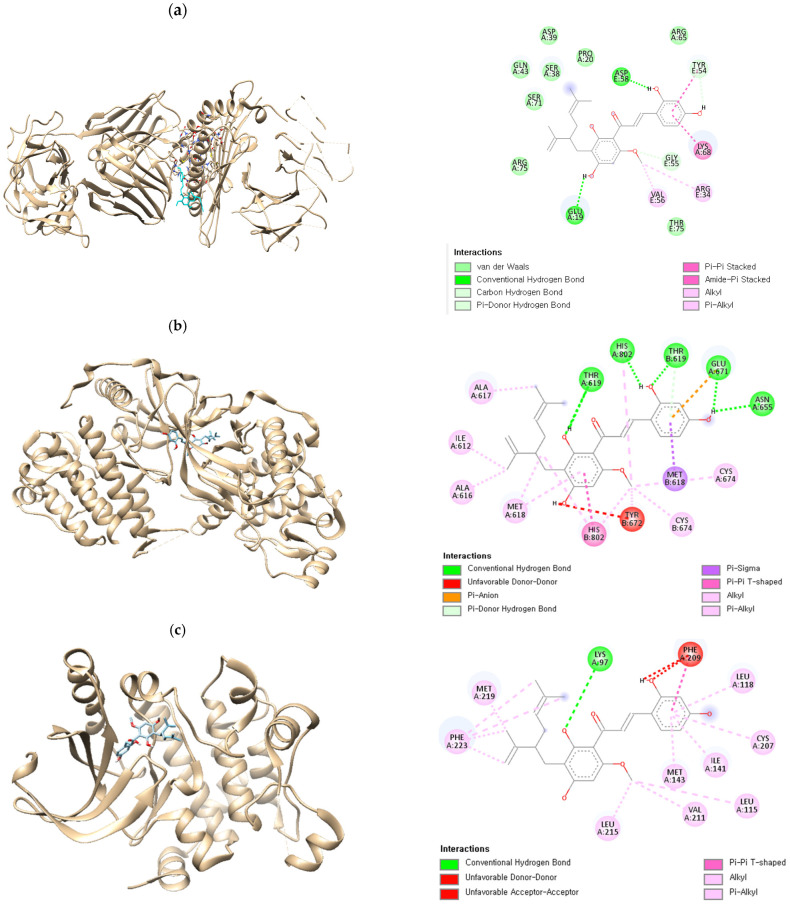

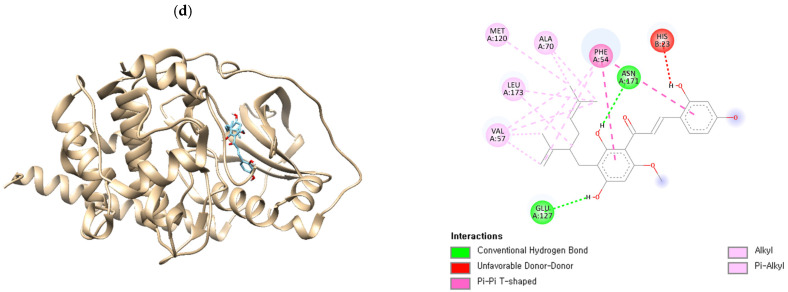
Molecular docking analysis of (**a**) kuraridin–tyrosinase, (**b**) kuraridin–c-KIT, (**c**) kuraridin–ERK1/2, and (**d**) kuraridin–PKC.

A molecular docking study was performed to verify the interaction between kuraridin and melanogenesis target genes screened by network pharmacology and to explore the molecular mechanism of the compound in hyperpigmentation. As shown in Figure 7, all four complexes between kuraridin and tyrosinase, c-KIT, ERK1/2, and PKC exerted strong interactions with the lowest binding energies of −8.4, −9.2, −9.2, and −7.5 kcal/mol, respectively. In general, the lower the binding energy, the more likely the ligand is to interact with the protein, and the ligands with a binding energy of less than −7.0 kcal/mol are considered to have high binding affinity for the target protein. [19]. In addition, various types of non-covalent interactions, such as hydrogen bonding and pi interactions, are present, resulting in the formation of tight and stable protein–ligand complexes after docking.

## 3. Discussion

Although proper melanin biosynthesis protects the skin from UV-induced DNA damage, abnormal melanin deposition after UV exposure leads to various skin hyperpigmentation disorders, including senile lentigo, post-inflammatory pigmentary alterations, and melasma [20]. Tyrosinase is a common target for anti-melanogenic agents because it is located in the rate-limiting step of melanin synthesis and has high targeting specificity with a low risk of side effects. Recently, natural anti-melanogenic agents have become essential alternatives to synthetic agents for cosmetic and medicinal applications because of their lower toxicity and better bioavailability.

In the search for effective melanogenic inhibitors from natural products, prenylated flavonoids, including kuraridin, kushenol A, and kurarinol from *S. flavescens* were evaluated for their anti-melanogenic activity via tyrosinase. Kuraridin is a prenylated chalcone compound with three functional groups, namely C8 lavandulyl, C5 methoxyl moieties of the A-ring, and 2′,4′-dihydroxyl moiety of the B-ring. Both kushenol A and kurarinol have functional groups similar to those of kuraridin; however, their backbone is a flavanone. Kuraridin (IC_50_, 0.16 μM) showed 392- to 543-fold higher activity than kurarinol and kushenol A in the anti-monophenolase activity of tyrosinase using _L_-tyrosine. In addition, the diphenolase activity of tyrosinase using _L_-DOPA was significantly inhibited only by kuraridin (IC_50_, 0.04 μM), demonstrating that the presence of the chalcone structure, the opening of the C-ring between C1 and C2, and four hydroxy moieties played a crucial role in determining the tyrosinase inhibitory activity.

Kuraridin, which proved to be the most potent tyrosinase inhibitor in the present study, was selected to elucidate its anti-melanogenic effects at the melanocyte level. As a result, pretreatment of the compound markedly reduced both intracellular and extracellular melanin levels by suppressing tyrosinase activity in *α*-MSH-induced B16F10 melanoma cells, demonstrating that its cellular tyrosinase inhibition was responsible for anti-melanogenic potential. It also reduced melanin synthesis in the basal epidermal layer of a 3D human skin model. As melanogenesis involves multiple processes, the anti-melanogenic mechanisms involved in kuraridin-mediated gene expression were worthy of further investigation.

Network pharmacology was used to elucidate the multiple target genes of kuraridin and its multidirectional regulation of melanogenic signaling pathways to discover effective lead compounds. Among the top ten hub genes for kuraridin, KIT, PRKCA, and MAP2K1 were strongly associated with the anti-melanogenic effects of kuraridin. Recent studies have demonstrated the critical role of the c-KIT signaling pathway encoded by the KIT gene, which is responsible for stimulating tyrosinase activity, resulting in receptor autophosphorylation to initiate signal transduction during melanogenesis [21,22]. These reports strongly support our in vitro results on the tyrosinase inhibitory effects of kuraridin by demonstrating the implications of c-KIT expression and downstream signaling pathways in processes related to tyrosinase activity. Moreover, the encoded c-KIT binds to SCF, which stimulates the ERK1/2 signaling pathway in melanogenesis, involving MAP2K1, another hub gene of kuraridin. Previous experimental studies have also demonstrated that ERK directly phosphorylates MITF, leading to its ubiquitin-dependent degradation [23]. Overall, the anti-melanogenic mechanism of kuraridin was found to be closely related to the inhibition of tyrosinase transcription and activity through two upstream signaling pathways. Kuraridin can be used as a naturally potent anti-melanogenic agent in the cosmetic and pharmaceutical industries.

Although further in vivo studies are required to demonstrate KIT, PRKCA, and MAP2K1 as the major potential anti-melanogenic targets of kuraridin, it has been shown that KIT and MAP2K1 are simultaneously involved in the c-KIT cascade, which is considered the main target of melanogenesis, and that PRKCA also acts directly at the transcriptional level of MITF and its downstream target melanogenic enzymes through another pathway.

## 4. Materials and Methods

### 4.1. Chemicals

Kuraridin (>98% purity) was purchased from BioBioPha Co. Ltd. (Kunming, China). Kushenol A (>98% purity) and kurarinol (>98% purity) were purchased from ChemFaces (Wuhan, China). Kojic acid (≥98.5% purity), tyrosinase, 3,4-dihydroxy-L-phenylalanine (L-DOPA), L-tyrosine, and α-MSH were purchased from Sigma-Aldrich (St. Louis, MO, USA). Dulbecco’s modified Eagle’s medium (DMEM) was purchased from Gibco (Grand Island, NY, USA). Phosphate-buffered saline (PBS) was purchased from Welgene (Daegu, Republic of Korea). Fetal bovine serum (FBS) and penicillin/streptomycin were purchased from HyClone Laboratories (Logan, UT, USA).

### 4.2. Tyrosinase Activity Assay

In the present study, purified tyrosinase with 6540 units/mg of activity was used. Tyrosinase from *Agaricus bisporus* is widely used as an established model for the discovery of novel tyrosinase inhibitors because its accessibility and inhibitory molecules show good potency against human and murine tyrosinases [24]. L-tyrosine was used as a substrate for the tyrosinase monophenolase activity assay, and L-DOPA was used as a substrate for the tyrosinase diphenolase activity assays. Briefly, kuraridin, kushenol A, kurarinol, and kojic acid were dissolved in dimethyl sulfoxide (DMSO) at 10 or 1 mM concentrations and diluted for in vitro dosage experiments. Kuraridin (0.01–0.2 µM), kushenol A (5–100 µM), kurarinol (5–100 µM), and kojic acid (5–100 µM) were mixed, in each case, with 100 μL of the aqueous solution of tyrosinase (200 units/mL) in a 96-well plate and pre-incubated at 25 °C. Then, 1.0 mM L-DOPA solution or 2.0 mM L-tyrosine was added to the mixture and incubated. Optical absorbance was measured at 475 nm.

Inhibitory activity rate (%) = 100 × [(Aa − Ab)/(Ac)] × 100

where Aa is the absorbance of the test sample and enzyme, Ab is the absorbance of the test sample without the enzyme, and Ac is the absorbance of the enzyme without the test sample.

### 4.3. Kinetic Analysis

Enzyme kinetics were analyzed using Lineweaver–Burk and Dixon reciprocal plots of the reaction rate and substrate concentration to evaluate the effect of the sample on the affinity of the substrate and enzyme. The tyrosinase concentration was kept constant at 200 U/mL, whereas the substrate (L-tyrosine/L-DOPA) concentration varied between 0.5 and 2.0 mM. The reaction was similar to the anti-tyrosinase assay described above; 100 µL of sample solution was added, followed by 100 µL of tyrosinase (200 units/mL) after incubation at 25 °C. The substrate was then added to the wells and mixed thoroughly, and kinetic measurements of the solution were performed at a wavelength of 475 nm.

### 4.4. In Silico Skin Bioavailability and Molecular Docking Simulation

The simplified molecular input line entry system (SMILES) format of kuraridin, kushenol A, and kurarinol from PubChem was entered into the pkCSM (https://biosig.lab.uq.edu.au/pkcsm/ (accessed on 5 April 2024)) and Protox 3.0 (https://tox.charite.de/protox3/ (accessed on 5 April 2024)) to assess pharmacokinetic and toxicity profiles [25].

The X-ray crystallographic structures of tyrosinase, c-KIT, ERK1/2, and PKC were obtained from the RCSB (Protein Data Bank ID: 7RK7, 6GQJ, 3PP1, and 4RA4, respectively) for docking simulations. The kuraridin structure was determined using the PubChem database (CID 44428631). The docking results were obtained from the conformation with the lowest free energy.

### 4.5. In Silico Network Pharmacology Analysis

The simplified molecular input line entry system (SMILES) structure of the compound was obtained from PubChem (https://pubchem.ncbi.nlm.nih.gov/ (accessed on 6 April 2024)). The structure was then imported into SwissTargetPrediction (https://bio.tools/SwissTargetPrediction (accessed on 10 April 2024)) database to predict compound-related target genes. The melanogenesis-related targets were retrieved from GeneCards (https://www.genecards.org/ (accessed on 15 April 2024)).

The interactions between kuraridin and the anti-melanogenic targets were analyzed using the STRING database (https://string-db.org/ (accessed on 30 April 2024)) and Cytoscape 3.10. Gene Ontology (GO) analysis and Kyoto Encyclopedia of Genes and Genomes (KEGG) pathway enrichment analyses were performed using the DAVID database (https://david.ncifcrf.gov/ (accessed on 30 April 2024)).

### 4.6. Cell Culture and Cell Viability

Murine B16F10 melanoma cells (Korean Cell Line Bank, Seoul, Republic of Korea) were cultured in DMEM containing 10% FBS and 1% penicillin-streptomycin at 37 °C in the humidified atmosphere of a 5% CO_2_ incubator. The cells were cultured until 100% confluent for all experiments.

Cell viability was assessed using the CCK-8 kit. Cells were incubated in 96-well plates at a density of approximately 3 × 10^3^ cells/well. After 24 h incubation, a kuraridin concentration (0.1–10 µM) of samples or medium was added to B16F10 cells at 72 h of incubation. Absorbance was monitored at 450 nm using a microplate reader (Synergy H(1), Biotek, VT, USA).

### 4.7. In Cellular Melanin Content Analysis

B16F10 melanoma cells (7.4 × 10⁴ cells/well) were seeded in a 6-well plate. The cells were then pretreated with kuraridin (0.1–5 μM) or kojic acid (500 μM) for 2 h, followed by stimulation with 0.1 μM of α-MSH for a further 72 h and cell harvest by trypsinization. The extracellular melanin content was measured by direct estimation of the absorbance of the cell-free culture medium at 405 nm. For the measurement of intracellular melanin content, cell pellets were dissolved in 1 N NaOH at 95 °C for 1 h. The absorbance of the resulting solution was measured at 405 nm using a microplate reader [Synergy H(1)].

### 4.8. Evaluation of Cellular Tyrosinase Activity

Tyrosinase activity was estimated by measuring the rate of L-DOPA oxidation. B16F10 cells (7.4 × 10⁴ cells/well) were incubated in a 6-well culture plate for 24 h with kuraridin (0.1–5 μM) or kojic acid (500 µM) for 2 h, followed by α-MSH (100 nM) treatment with culture for 72 h. The cells were washed with cold PBS, and cell lysis buffer was added for lysis at 4 °C for 1 h on ice and centrifuged at 13,000 rpm for 20 min at −4 °C to obtain supernatants. Protein concentration was quantified at 50 µg using a BCA protein assay kit. L-DOPA (10 mM) was added to the quantified protein and incubated at 37 °C for 1 h. Absorbance was measured at 490 nm using a microplate reader.

### 4.9. Ex Vivo 3D Pigmented Human Skin Model

Neoderm^®^-ME is a 3D model containing human primary keratinocytes and melanocytes that mimics human skin. The human skin model was transferred to a 12-well plate in maintenance medium (Tego Science, Seoul, Republic of Korea) containing kuraridin (0.1, 1, 5 μM) and incubated at 37 °C in 5% CO_2_ for 7 days. The medium was changed once a day. After 7 days, Fontana–Masson staining was performed to quantify melanin content.

### 4.10. Statistical Analysis

The analysis results are expressed as the mean ± standard deviation (SD) and run in triplicates (*n* = 3). Statistical analysis was performed using Duncan’s multiple range test with the Statistical Analysis System (SAS) version 9.3 (SAS Institute, Cary, NC, USA) for multiple comparisons.

## 5. Conclusions

The present study confirmed that kuraridin exhibited the most remarkable inhibitory effect on tyrosinase activity in cell-free and α-MSH-induced melanoma cells. It effectively reduced melanin content in melanoma and a reconstituted 3D human skin model. The KIT, PRKCA, and MAP2K1 genes were identified as crucial targets for kuraridin, and the critical roles of the compound in the c-KIT and ETB-R signaling pathways involving the melanogenic upstream cascade through the regulation of the three genes were elucidated based on network pharmacology. Moreover, molecular docking results showed that kuraridin formed highly stable protein–ligand complexes with the proteins encoded by three target genes. Although more extensive experimental validation in clinical trials is required, these results are expected to provide a novel mechanism of action for kuraridin against melanogenesis and its potential use as a promising source of skin-whitening cosmetics or depigmentation agents for hyperpigmentation disorders.

## Figures and Tables

**Table 1:** Inhibitory effects of compounds kuraridin, kushenol A, and kurarinol on tyrosinase activities.

Compound	IC_50_ (µM)		Ki (µM)		Inhibition Type	
	_L_-Tyrosine	_L_-DOPA	_L_-Tyrosine	_L_-DOPA	_L_-Tyrosine	_L_-DOPA
Kuraridin	0.16	0.04	0.24	0.33	Non-competitive	Competitive
Kushenol A	62.70	136.10	102.30	-	Non-competitive	-
Kurarinol	86.92	205.30	100.60	-	Non-competitive	-
Kojic acid ^1^	23.73	21.25	1.10 ^2^	3.60 ^2^	Mixed ^2^	Mixed ^2^

^1^ Positive control; ^2^ Deri et al. (2016) [15].

**Table 2:** In silico, pharmacokinetic profiles of kuraridin, kurarinol, and kushenol A were determined using pkCSM and Protox 3.0.

Property	Samples			Desired Value
	Kuraridin	Kushenol A	Kurarinol	
Human intestinal absorption (%)	73.66	90.12	79.35	<30% (poorly absorbed)
Skin permeability (Log Kp)	−2.73	−2.74	−2.73	Low skin permeability > −2.5
Minnow toxicity	0.81	0.88	2.02	High acute toxicity < −0.3
Skin sensitization	No	No	No	No
Mutagenicity	No	No	No	No
Hepatotoxicity	No	No	No	No
Carcinogenicity	No	No	No	No

**Table 3:** Identification of top 10 hub genes in kuraridin action.

Genes	Degree	Average Shortest Path Length	Betweenness Centrality	Closeness Centrality
PPARG	12	1.333	0.255	0.750
PTGS2	11	1.444	0.193	0.692
KIT	9	1.500	0.180	0.667
PARP1	9	1.667	0.040	0.600
PRKCA	8	1.611	0.205	0.621
TERT	7	1.722	0.027	0.581
CDK2	6	1.833	0.005	0.546
CDK4	6	1.889	0.003	0.529
MAP2K1	6	1.889	0.008	0.529
PPARA	5	1.833	0.027	0.546

## Data Availability

The data presented in this study are available on request from the corresponding author. The data are not publicly available since they are raw data.
